# Caregiver burden in owners of dogs and cats undergoing anticancer therapy in a referral hospital in Hong Kong

**DOI:** 10.1111/jsap.70015

**Published:** 2025-08-14

**Authors:** K. M. Tam, A. Giuliano, S. S. U. H. Bukhari, P. V. Steagall

**Affiliations:** ^1^ Jockey Club College of Veterinary Medicine and Life Science City University of Hong Kong Hong Kong SAR China; ^2^ Department of Veterinary Clinical Sciences, Jockey Club College of Veterinary Medicine and Life Sciences City University of Hong Kong Hong Kong SAR China; ^3^ CityU Veterinary Medical Centre City University of Hong Kong Hong Kong SAR China; ^4^ Centre for Animal Health and Welfare, Jockey Club College of Veterinary Medicine and Life Science City University of Hong Kong Hong Kong SAR China

## Abstract

**Objectives:**

To assess the prevalence and factors associated with increased caregiver burden among owners of dogs and cats undergoing anti‐cancer therapy in Hong Kong.

**Materials and Methods:**

Clients of the oncology service of a large veterinary hospital in Hong Kong completed an online survey during real‐time consultations. Caregiver burden was based on an adapted and abbreviated Zarit Burden Interview score (seven questions; up to 28 points with cut‐off of ≥9 points for increased burden). Client and patient demographics, along with oncological treatment data, were collected. Data were analysed using the chi‐square (*χ* ^2^) test and odds ratios, with P < 0.05 considered significant.

**Results:**

Of the 54 responses, 70.4% of caregivers reported increased burden. Caregivers not working in the human medical field experienced an increased burden compared to those working in the field (P = 0.03, *χ* ^2^ = 4.26, odds ratio = 7.54). Increased burden was associated with caregivers’ concerns about the cost of treatment (P = 0.01, *χ* ^2^ = 11.96, odds ratio = 2.30) and its potential side effects (P = 0.03, *χ* ^2^ = 10.27, odds ratio = 12.24). Caregivers reported an increased burden with prolonged treatment durations (P = 0.01, *χ* ^2^ = 14.17, odds ratio = 2.39). The burden level was not significantly different between cat and dog caregivers (P = 0.27).

**Clinical Significance:**

Caregiver burden is observed in owners of dogs and cats undergoing anticancer therapy affecting their psychosocial well‐being. Recognising factors associated with increased burden enables veterinarians to provide better support to caregivers and improve veterinarian‐client relationships.

## INTRODUCTION

Caregiver burden describes the stress experienced by individuals who care for a sick family member (Zarit et al., 1980). This stress is the result of both the objective aspect of providing care, including time, money and physical demands, as well as the subjective perception and emotions of the caregiver (Tremont, 2011). While caregiver burden in humans has been well‐studied (Cham et al., 2022; Pinquart & Sörensen, 2003; Secinti et al., 2023), research on pet caregiver burden is recent, with the earliest qualitative study dating back to 2013 (Christiansen et al., 2013). Subsequently, more compelling evidence has emerged and large cross‐sectional observational studies have been conducted (Spitznagel et al., 2017, 2023; Spitznagel, Patrick, Hillier, et al., 2022). Indeed, burden has been consistently demonstrated among caregivers of animals with chronic or terminal disease (Silva et al., 2024; Spitznagel et al., 2017), dermatological (Silva et al., 2024; Spitznagel et al., 2021, 2023; Spitznagel, Solc, et al., 2019), behavioral (Kuntz et al., 2023) and cognitive disorders (Taylor et al., 2024) as well as osteoarthritis (Spitznagel, Patrick, Gober, et al., 2022).

Caregiver burden has gained significant attention in veterinary medicine, primarily due to its association with poor psychosocial outcomes among pet owners and its effect on veterinarian well‐being. A high burden has been consistently linked to elevated stress, symptoms of anxiety and depression and poor quality of life (Shaevitz et al., 2020; Spitznagel et al., 2017). Furthermore, caregiver burden potentially impacts the veterinary‐client relationship via burden transfer, which involves the shifting of burden and stress from clients to veterinarians through stressful encounters (Spitznagel, Ben‐Porath, et al., 2019). This burden transfer has the potential to contribute to higher levels of stress and burnout among veterinarians (Spitznagel, Ben‐Porath, et al., 2019), which is a common phenomenon in the veterinary profession (Ouedraogo et al., 2021).

The recognition of caregiver burden in a timely manner is crucial to prevent its escalation and mitigate the negative impacts on the well‐being of both pet owners and veterinarians. As cancer is one of the most common chronic illnesses in veterinary patients, it is important to investigate caregiver burden in these cases. Caregiver burden has been found in suspected cancer patients (Shaevitz et al., 2020) and pets undergoing oncological treatment (Silva et al., 2024). However, the predictors of burden related to anticancer therapy have not been thoroughly examined. Identifying potential risk factors may allow the veterinary team to intervene and address the burden at an early stage.

This study hypothesised that caregiver and patient demographics (e.g. caregiver age, gender, total number of pets, duration of illness, cancer and treatment types) would affect the degree of caregiver burden. The aim of this study was to assess the prevalence and factors associated with caregiver burden among owners of cats and dogs undergoing anticancer therapy.

## MATERIALS AND METHODS

### Study design

The caregiver burden was examined using a cross‐sectional closed survey by convenience sampling in this study. Data were collected in Hong Kong between November 2023 and August 2024. Caregivers of dogs and cats presented to the oncology service were invited to participate and complete an online questionnaire. Inclusion criteria included pet owners who acted as caregivers for their animals, were at least 18 years old, and could understand written English and/or traditional or simplified Chinese. Exclusion criteria involved first consultations or patients who had not started anticancer therapy yet. Participation in the study was voluntary and did not include any financial compensation. Written informed consent was obtained from all caregivers. Details about the study, including objectives, eligibility criteria for participation, participants’ rights regarding confidentiality and privacy, and data protection, were listed in the consent. Any identifiable information was accessed by authorised personnel and was used in accordance with the provisions of the Personal Data Privacy Ordinance of Hong Kong (Lai‐ling & Guobin, 2024).

The participating caregivers completed an online questionnaire using an electronic tablet, while the medical records of their pets were carefully reviewed. The review included details about species, age, cancer diagnosis, duration and types of anticancer treatments, and any existing comorbidities. Data were analysed and compared between individuals with increased versus normal burden according to the Zarit Burden Interview score (Spitznagel, Mueller, et al., 2019). This research was reviewed and approved by the Human Subject Ethics Sub‐Committee, City University of Hong Kong, with the approval reference: [HU‐STA‐00000663], 23rd October 2023.

### Questionnaire design

The questionnaire included questions assessing the caregiver demographics, the level of caregiver burden and the perceived challenges associated with anticancer therapy. The initial survey in English was translated and back‐translated to ensure accurate semantics in traditional and simplified Chinese. The expected time to finish the questionnaire was between 5 and 10 minutes. The first section of the questionnaire aimed to collect information about the caregiver’s age and gender, total number of pets and number of co‐caregivers. It also inquired about the caregiver’s experience with pet ownership and caring for sick pets, any medical background and the duration of the animal’s illness. The second section included an abbreviated version of the adapted Zarit Burden Interview (ZBI). The original ZBI was developed to assess the burden experienced by human caregivers (Zarit et al., 1980), which was later modified and abbreviated to seven questions to investigate the burden in pet owners (Spitznagel et al., 2017; Spitznagel, Mueller, et al., 2019). Respondents could choose from options ranging from “never” to “nearly always”. Each response was assigned a numerical value (i.e. “never” = 0; “nearly always” = 4). The cumulative score represents the level of burden, with a cut‐off point of ≥9 indicating clinically meaningful increased caregiver burden (Spitznagel, Mueller, et al., 2019). The third section assessed caregivers’ agreement with statements about their perceived concerns related to providing anticancer therapy. These concerns included cost, potential side effects, time constraints, treatment effectiveness and safety issues associated with chemotherapy handling. The agreement was evaluated using Likert‐style answers, with 1 indicating “strongly disagree” and 5 indicating “strongly agree”. Respondents were able to review and change their answer or terminate the survey before submission. Time stamps were used to identify duplicated answers. The full questionnaire is presented in the supporting information. Variables collected during the study are presented in Table 1.

**Table 1:** Variables collected during the study

Variable collected	Assigned short name	Variable structure
Age of caregiver (years)	Caregiver age	Categorical
Gender	Caregiver gender	Binary – yes or no
Species of your pet	Species	Binary – cat or dog
Sex of your pet	Pet sex	Categorical
Age of pet	Pet age	Continuous
Adapted Zarit Burden Interview (ZBI) score	ZBI score	Continuous
Number of people caring for the pet at home?	Help from others	Binary – only me or two or more
Number of pet(s) under the care of pet caregiver?	Number of pets	Categorical
First time to have pet?	First time pet owner	Binary – yes or no
Experience of caregiving for sick animals before?	Experience in caring for sick animals	Binary – yes or no
Work in the field of human health?	Work in human medical field	Binary – yes or no
Duration of pet sickness	Duration of sickness	Categorical
Types of cancer diagnosed	Types of cancer	Categorical
Presence of cancer metastasis	Metastasis	Binary – yes or no
Presence of any comorbidity	Comorbidity	Binary – yes or no
Modality of anticancer therapy	Modality	Binary – yes or no
Types of anticancer therapy being used	Types of anticancer therapy	Categorical
Duration of treatment	Duration of treatment	Categorical
I feel stressed about the time spent on providing anticancer therapy to my pet	Concern about time spent	Categorical
I am worried about the potential side effects of drugs on my pet	Potential side effects	Categorical
I am concerned about the cost of the anticancer therapy	Cost	Categorical
I am uncertain about the effectiveness of the anticancer therapy on my pet	Effectiveness	Categorical
I feel stressed about giving the treatment to my pet on time	Punctuality for appointments	Categorical
I feel stressed about the special handling of my pet’s body secretions after chemotherapy (e.g. saliva, urine and faeces)	Special handling of drug	Categorical
Has your overall stress level changed with your pet’s anticancer therapy?	Overall stress	Categorical

Each variable was assigned a short name, with a corresponding structure (binary, categorical)

### Statistical analysis

All statistical analyses were carried out using RStudio with R version 4.2.3. Continuous data (age and ZBI scores) were presented as mean ± SD. Categorical data were described as frequency and percentage. An unpaired *t*‐test compared the means of ZBI scores between cat and dog caregivers. Caregivers were divided into either “normal” or “increased” burden based on the above ZBI cut‐off score (Spitznagel, Mueller, et al., 2019). The two levels of caregiver burden were compared with all the variables in the questionnaire using the Chi‐Square test. The outcomes of the Chi‐Square test were presented as Chi‐Square value (*χ* ^2^ value), degree of freedom (df) and P‐value. Odds ratios (ORs) and their 95% confidence intervals (95% CI) were calculated for categorical variables that showed significant associations (P < 0.05) with caregiver burden.

## RESULTS

### Demographic characteristics of pet owners and animals

Fifty‐five pet caregivers met the inclusion criteria and were invited to participate in the study; 54 of whom completed the questionnaire for a total completion rate of 98%. The incomplete response was excluded from further data analysis. Table 2 shows caregiver and patient demographics.

**Table 2:** Caregiver and patient demographics as well as type of cancer, duration of treatment and sickness

Variable	Categories	Number	Percentage
Caregiver gender	Female	40	74.1
	Male	14	25.9
Caregiver age (years)	18 to 24	1	1.9
	25 to 34	2	3.7
	35 to 44	18	33.3
	45 to 54	16	29.6
	55 to 64	13	24.2
	More than 65	4	7.3
Species	Cat	14	25.9
	Dog	40	74.1
Pet sex	Male entire	4	7.4
	Male neutered	11	20.4
	Female entire	15	27.8
	Female spayed	24	44.4
Number of pets	One	24	44.5
	Two	14	25.9
	Three or more	16	29.6
First time pet owner	Yes	24	44.5
	No	30	55.5
Help from other	Only me	8	14.8
	Two or more	46	85.2
Work in human medical field	Yes	4	7.3
	No	50	92.7
Experience in caring for sick animals	Yes	23	42.6
	No	31	57.4
Duration of sickness	<1 month	4	7.3
	1 to 3 months	15	27.8
	4 to 6 months	10	18.3
	7 to 12 months	5	9.3
	More than a year	20	37.0
Duration of treatment	One off	2	3.7
	Less than a month	17	31.4
	1 to 3 months	13	24.1
	4 to 6 months	5	9.3
	7 to 12 months	5	9.3
	More than a year	12	22.2
Types of cancer	Adenocarcinoma	1	1.9
	Anal sac adenocarcinoma	3	5.5
	Carcinoma	5	9.3
	Hemangiosarcoma	1	1.9
	Histiocytic sarcoma	2	3.8
	Leukaemia	1	1.9
	Lymphoma	18	33.3
	Mast cell tumour	5	9.3
	Melanoma	5	9.3
	Osteosarcoma	3	5.5
	Multiple myeloma	1	1.9
	Soft tissue sarcoma	3	5.5
	Urothelial carcinoma	6	11.1

### Cancer and anticancer therapy

Most animals had lymphoma (*n* = 18, 33.3%; Table 2). Among the 44 animals that underwent staging, 36.3% were suspected to have metastasis. The majority of animals (*n* = 43, 79.6%) did not have any comorbidities. A total of 64.9% (*n* = 35) received unimodal anticancer treatment, while the remaining (*n* = 19, 35.1%) underwent multimodal therapy. Among those receiving unimodal treatment, chemotherapy was the most common (*n* = 24, 44.4%; Table 3).

**Table 3:** Types of anticancer therapies administered in this study

Variable	Categories	Modality	Number	Percentage
Types of anticancer therapy	Surgery	Unimodal	2	3.7
	Chemotherapy	Unimodal	24	44.5
	Target therapy	Unimodal	8	14.8
	Immunotherapy	Unimodal	1	1.9
	Surgery and chemotherapy	Multimodal	4	7.3
	Surgery and target therapy	Multimodal	2	3.7
	Surgery and immunotherapy	Multimodal	3	5.5
	Chemotherapy and target therapy	Multimodal	3	5.5
	Chemotherapy and electrochemotherapy	Multimodal	1	1.9
	Surgery, chemotherapy and target therapy	Multimodal	3	5.5
	Surgery, chemotherapy and immunotherapy	Multimodal	1	1.9
	Target therapy, ration and electrochemotherapy	Multimodal	1	1.9
	Surgery, chemotherapy, immunotherapy and electrochemotherapy	Multimodal	1	1.9

### Owners’ perceived challenges from anticancer therapy

More than two‐thirds of pet caregivers expressed concerns about the potential side effects of drugs (*n* = 42, 77.8%) and the cost of treatment (*n* = 37, 68.5%). Most participants did not express concerns regarding the special handling of chemotherapeutic drugs (*n* = 35, 64.8%) or punctuality for appointments (*n* = 34, 62.9%). In terms of perceived changes in overall stress during anticancer therapy, 59.3% (*n* = 32) reported an increase in stress, while 24.1% (*n* = 13) reported no change and 16.7% (*n* = 9) reported a decrease (Table S1).

### ZBI score and caregiver burden

The overall ZBI score was 9.8 ± 3.6. Most caregivers (*n* = 38, 70.4%) experienced an increased burden, whereas 29.6% (*n* = 16) had a normal burden. The ZBI scores for cat and dog owners were 10.8 ± 2 and 9.5 ± 3.9, respectively. There was no significant difference between cat and dog ZBI scores (P = 0.27). The numbers and percentages for categorical responses regarding the caregiver burden ZBI scores are presented in Table 4.

**Table 4:** Numbers and percentages for categorical responses related to the estimations of the Adapted Zarit Burden Interview (ZBI) scores

Variable	Categories	Number	Percentage
Do you feel that because of the time you spend with your pet that you don’t have enough time for yourself?	Never	8	14.8
	Rarely	13	24.1
	Sometimes	21	38.9
	Quite frequently	3	5.6
	Nearly always	9	16.6
Do you feel stressed between caring for your pet and trying to meet other responsibilities for your family or work?	Never	2	3.7
	Rarely	15	27.8
	Sometimes	27	50.0
	Quite frequently	9	16.6
	Nearly always	1	1.9
Do you feel you have lost control of your life since your pet’s illness?	Never	11	20.4
	Rarely	26	48.0
	Sometimes	11	20.4
	Quite frequently	3	5.6
	Nearly always	3	5.6
Do you feel angry when you’re around your pet?	Never	40	74.1
	Rarely	12	22.2
	Sometimes	2	3.7
	Quite frequently	0	0.0
	Nearly always	0	0.0
Do you feel embarrassed over your pet’s behaviour?	Never	39	72.2
	Rarely	13	24.1
	Sometimes	2	3.7
	Quite frequently	0	0.0
	Nearly always	0	0.0
Do you feel you should be doing more for your pet?	Never	5	9.3
	Rarely	8	14.8
	Sometimes	21	38.9
	Quite frequently	8	14.8
	Nearly always	12	22.2
Do you feel you could do a better job in caring for your pet?	Never	8	14.8
	Rarely	8	14.8
	Sometimes	25	46.3
	Quite frequently	5	9.3
	Nearly always	8	14.8

### Factors associated with increased caregiver burden

Caregivers who do not work in the human medical field experienced increased burden compared to those who do (P = 0.03, df = 1, *χ* ^2^ = 4.26, OR = 7.54; Table 5). Increased caregiver burden was observed in individuals who had concerns about the side effects of anticancer drugs (P = 0.03, df = 4, *χ* ^2^ = 10.27, OR = 12.24) and the cost of therapy (P = 0.01, df = 4, *χ* ^2^ = 11.96, OR = 2.30). Additionally, prolonged duration of treatment was associated with increased levels of burden (P = 0.01, df = 5, *χ* ^2^ = 14.17, OR = 2.39; Table 6). No other variables demonstrated a significant relationship with caregiver burden.

**Table 5:** Results of the Chi square (*χ* ^2^) test with respect to caregiver burden. Significant associations are indicated by bold P‐values (P < 0.05)

Variable	Chi‐square (*χ* ^2^)	df	P value
Caregiver gender	0.61	1	0.43
Caregiver age (years)	3.03	5	0.69
Species	0.61	1	0.43
Pet sex	1.71	3	0.63
Number of pets	1.61	2	0.44
First time pet owner	1.60	1	0.20
Help from other	0.27	1	0.59
Work in human medical field	4.26	1	**0.03**
Experience in caring for sick animals	0.01	1	0.91
Duration of sickness	3.24	4	0.51
Duration of treatment	14.17	5	**0.01**
Types (modality) of anticancer therapy	0.73	1	0.39
Concern about time spent	7.75	4	0.10
Concerns about potential side effects of anticancer drug	10.27	4	**0.03**
Concerns about cost of anticancer therapy	11.96	4	**0.01**
Effectiveness of anticancer therapy	3.85	4	0.42
Concerns about punctuality for appointments	2.90	4	0.57
Concerns about special handling of drug	3.47	4	0.74
Overall stress	6.13	4	0.19
Metastasis	3.39	2	0.18
Comorbidity	0.60	1	0.43

**Table 6:** Odds ratios and 95% confidence intervals (CI) of the categorical variables with respect to caregiver burden

Variable	Categories	Number	Odds ratio	95% CI lower	95% CI upper	P‐value
Work in the human medical field	Yes	4	1			0.03
	No	50	7.54	0.79	229.88	
Concerns about potential side effects of anticancer drug	Much decrease	2	1			0.03
	Slightly decrease	3	1	0.01	57.76	
	No changes	7	2.33	0.12	96.88	
	Slight increase	24	4.31	0.30	150.67	
	Much increase	18	12.24	0.72	492.29	
Concerns about cost of anticancer therapy	Much decrease	3	1			0.01
	Slightly decrease	1	0.57	0.008	38.34	
	No changes	13	0.35	0.009	5.42	
	Slight increase	20	2.78	0.07	46.18	
	Much increase	17	2.30	0.05	38.65	
Duration of treatment	One off	2	1			0.01
	Less than a month	17	1.24	0.03	18.91	
	1 to 3 months	13	1.67	0.04	28.55	
	4 to 6 months	5	1.82	0.03	96.11	
	7 to 12 months	5	0.14	0.002	3.23	
	More than a year	12	2.39	0.05	47.32	

## DISCUSSION

The study of caregiver burden is important because it describes the stress experienced by individuals who care for a sick family member (Zarit et al., 1980); therefore, it has profound implications for the veterinary‐client relationship, caregiver wellbeing and animal welfare. While previous studies found an increase in burden among caregivers in the United States of America and Brazil (Silva et al., 2024; Spitznagel et al., 2017), the present study is the first to report caregiver burden among pet caregivers in Hong Kong. More than two‐thirds of pet caregivers experienced an increased burden while caring for their sick animals undergoing anticancer therapy. The duration of treatment, concerns about treatment side effects and costs were significantly associated with increased levels of burden. Additionally, caregivers not working in the human medical field experienced a significantly increased burden compared to those working in the field. These findings underscore the need to address the specific challenges faced by caregivers of small animal patients undergoing anticancer therapy and to develop appropriate support systems to help alleviate their burden.

A recent study conducted in Brazil also reported an increased burden in over one‐third of caregivers of animals undergoing cancer treatment (Silva et al., 2024). In contrast, our study recorded a higher prevalence, potentially due to differences in demographics. Consistent with a recent study where a mean ZBI score of 12.98 was measured among owners of cancer‐bearing dogs (Leonardi et al., 2024), the present study also recorded a mean ZBI score of 9.80, in which both studies suggested an overall increase in caregiver burden. This is in contrast with another study where a mean of 5.92 was recorded in caregivers of cats and dogs with cancer (Spitznagel, Mueller, et al., 2019). This study did not report demographic data, which could explain the different mean ZBI scores and burden levels between studies. Another study, using the adapted ZBI with a cutoff score of 18 indicating meaningful burden, recorded a median score of 11 in owners of animals with suspected cancer, suggesting that the participants in this study experienced a lower overall burden (Shaevitz et al., 2020). One notable difference between the latter study and our study sample was the duration of illness. In that study, over 60% of the animals were sick for less than a month, whereas in our samples, more than 90% had been ill for over a month, with 65% having started anticancer therapy for more than a month in the present study.

Prolonged duration of treatment was associated with increased caregiver burden. Similarly, the Brazilian study also noted a positive correlation between therapy duration and degree of caregiver burden in owners of dogs and cats undergoing cancer treatment (Silva et al., 2024). It is reasonable to expect that with longer caregiving periods related to increased duration of treatment, there will be intense mental, physical and financial demands.

Cost of treatment was associated with increased caregiver burden. Our study measured the owners’ subjective perception of stress related to treatment costs rather than directly comparing the treatment cost with the owners’ income, evaluating the cost of each treatment itself or with other procedures and services (e.g. surgery and imaging). Caregivers who reported greater concern about treatment costs experienced increased burden than those who did not. According to the Veterinary Cancer Society, veterinary oncologist consultation fees alone cost about $125 to $250, while chemotherapy costs from $150 to $600 per dose and radiation therapy can cost up to $6000. Considering that most cancers are not curable and multiple treatments are often required to manage the disease progression, it is expected that the disease may cause long‐term financial burden. Previous research also demonstrated that increased caregiver burden is associated with financial strain (Britton et al., 2018). Another study found that the lower the income, the greater the burden caregivers experienced in dogs and cats undergoing cancer therapy (Silva et al., 2024). Caregivers with less income may not afford treatment costs, which will obviously lead to increased burden.

The potential side effects of anticancer therapy were associated with increased burden. A previous study indicated that more than half of pet caregivers would opt against chemotherapy due to the negative impact of the associated side effects. Indeed, less than one‐fifth of all respondents actually treated their animals with chemotherapy (Williams et al., 2017). Fear of side effects might originate from previous experiences of family members and friends, and this preconception might lead to reluctance to pursue cancer therapy (Kidd, 2008; Williams et al., 2017). On the other hand, another study assessing pet owners’ perception of radiation therapy found that after completion of treatment, owners became less concerned about the associated side effects of radiation therapy and repeated anesthesia (Smith et al., 2019), suggesting that much of the initial worry may be rooted in misconceptions and the lack of education. Other studies revealed that owners in general agreed cancer treatment provided the best possible outcome and would have taken the same decision again (Brønden et al., 2003; Mellanby et al., 2002). Contrary to our study, a recent study did not identify an association between owners’ perception of chemotherapy‐related adverse effects and owner‐reported measures of caregiver burden (Leonardi et al., 2024). Nonetheless, veterinarians should always provide a detailed explanation on treatment milestones, potential side effects and their likelihood to clients. Regular check‐ins provide support and mitigate side effects, which may address caregivers concerns and reduce burden.

Caregivers without medical backgrounds reported increased burden relative to those in the medical field. This study found that medical knowledge can be useful when caring for sick animals, as these individuals presented normal burden. The knowledge of medicine has a protective effect on caregiver burden, as higher levels of preparedness in human caregivers were associated with reduced levels of burden (Scherbring, 2002). Similarly, increased caregiver burden was linked to lower perceived health competence in informal caregivers of humans caring for family members with Alzheimer’s disease (Bailes et al., 2016). Medical background may help pet owners in understanding underlying disease processes and the effects of anticancer therapy. This could also reflect better education to handle expectations, treatment side effects and milestones. Previously, a brief educational intervention alleviated stressful conditions experienced by family caregivers of human heart attack patients (Abbasi et al., 2025). This finding highlights that empowering owners to be more knowledgeable about the disease process and treatment may help lower their burden.

Contrarily to our study, previous work found a lower burden among caregivers of sick cats than those of dogs (Spitznagel et al., 2023). This may be because of differences in disease types: animals in our study had only cancer, whereas the animals from the previous study had a variety of conditions, such as cancer and kidney, skin and/or gastrointestinal diseases. The variety of diseases, and therefore their treatment types, costs and duration may account for the observed differences.

Caregiver burden carries important implications. It has been increasingly identified in practice (Kuntz et al., 2023; Silva et al., 2024; Spitznagel et al., 2017, 2021, 2023; Spitznagel, Patrick, Gober, et al., 2022; Spitznagel, Patrick, Hillier, et al., 2022; Spitznagel, Solc, et al., 2019; Taylor et al., 2024); veterinary teams should understand and acknowledge its negative effects on the psychosocial health of clients (Spitznagel et al., 2017) and veterinarians through burden transfer and burnout (Spitznagel, Ben‐Porath, et al., 2019), which might impact veterinary‐client relationships. Our findings may help veterinary health professionals in the field of oncology to be more aware of increased caregiver burden and provide guidance based on factors associated with increased caregiver burden. Interventions to reduce burden include goals of care conversations, treatment based on the spectrum of care, regular check‐ins and establishing partnerships with mental health professionals (Goldberg, 2017; Spitznagel & Carlson, 2019).

The present study has limitations. The survey did not ask if owners worked in animal medical fields, which could have been an interesting question to include, as working in the human medical field was associated with less burden. The limited sample size restricts the generalisation of the findings, highlighting the need for further data collection with larger and more diverse samples in Hong Kong and Asia. It is difficult to understand if our findings are related to cultural differences or how the veterinary profession is rapidly evolving. Moreover, the use of convenience sampling conducted at a single referral hospital may have introduced selection bias, as the population seeking anticancer therapy at this facility might not represent those seeking treatment at general practices and other specialist hospitals. It would be interesting to compare caregiver burden associated with different procedures and practices in several countries. Additionally, our study did not investigate the burden associated with some anti‐cancer therapies such as radiation therapy. It is possible that our findings may have underrepresented clients who opted for radiation therapy, potentially leading to different experiences. Therefore, conducting a future multi‐center study may provide more insight into caregiver burden across different treatment types. The abbreviated ZBI is a validated tool that correlated well with its full version in terms of measurement properties. However, the tool was validated among the US population (Spitznagel, Mueller, et al., 2019). There might be inherent cultural differences between the Hong Kong and US populations that affect the experience of caregiving and the psychosocial function of caregiver burden. For instance, the presence of paid domestic helpers, a unique cultural feature in Hong Kong (He & Wu, 2019), might influence the sharing of caregiving responsibilities. While the current study investigated shared caregiving, paid domestic helpers were not specifically addressed.

## CONCLUSIONS

The majority of pet caregivers undergoing anticancer therapy experienced increased burden. Notably, caregivers not working in the human medical field reported an increased burden, as did those who expressed concerns about treatment costs and potential side effects of anticancer therapy. Additionally, caregivers experienced an increased burden associated with longer treatment durations. These findings underscore the need for targeted support and interventions aimed at alleviating caregiver burden. This may help foster a more supportive environment for pet caregivers, improving the veterinary‐client relationship with an ultimate benefit to animal welfare.

## Author contributions

K. M. Tam: Writing – original draft, conceptualisation, investigation, and methodology. A. Giuliano: Data curation, investigation and supervision. S. S. U. H. Bukhari: Writing – review and editing, data curation, and formal statistical analysis. P. V. Steagall: Writing – review and editing, conceptualisation, methodology and supervision.

## Funding information

This research did not receive any specific grant from funding agencies in the public, commercial, or not‐for‐profit sectors.

## Conflict of interest

No conflicts of interest have been declared.

## Consent to participate

Informed consent was obtained from the participants before the start of data collection.

## Supporting information


Table S1.

## Data Availability

The data that support the findings of this study are available on request from the corresponding author.
