# Supplementary material for: Caregiver burden in owners of dogs and cats undergoing anticancer therapy in a referral hospital in Hong Kong
Source: J Small Anim Pract. 2025 Aug 14;67(1):59–66. doi: 10.1111/jsap.70015 (PMC12828097; doi:10.1111/jsap.70015)
Supplement: Supplementary file 1 — Table S1. [file JSAP-67-59-s001.docx]

**Title: Caregiver burden in owners of dogs and cats undergoing anticancer therapy in a referral hospital in Hong Kong**

**Study Questionnaire**

1. How old are you?

- 18-24 year old
- 25-34 year old
- 35-44 year old
- 45-54 year old
- 55-64 year old
- 65 year old or above

1. What is your gender?

- Female
- Male
- Other

1. How many people care for your pet at home?

- Only me
- Two or more

1. How many pet(s) are under your care at the moment?

- 1
- 2
- 3 or more

1. Is this your first time you have your own pet?

- Yes
- No

1. Do you have experience of caregiving for sick animals before this pet?

- Yes
- No

1. Do you work in the field of human health?

- Yes
- No

1. How long has your pet been sick?

- < 1 month
- 1-3 months
- 4-6 months
- 7-12 months
- More than a year

**The following question will help estimate your burden level (Adapted Zarit Burden Interview (ZBI) score).**

| 9. Do you feel that because of the time you spend with your pet that you don't have enough time for yourself? | Never | Rarely | Sometimes | Quite frequently | Nearly Always |
| --- | --- | --- | --- | --- | --- |
| 10. Do you feel stressed between caring for your pet and trying to meet other responsibilities for your family or work? | Never | Rarely | Sometimes | Quite frequently | Nearly Always |
| 11. Do you feel that you have lost control of your life since your pet illness? | Never | Rarely | Sometimes | Quite frequently | Nearly Always |
| 12. Do you feel angry when you're around your pet? | Never | Rarely | Sometimes | Quite frequently | Nearly Always |
| 13. Do you feel embarrassed over your pet's behaviour? | Never | Rarely | Sometimes | Quite frequently | Nearly Always |
| 14. Do you feel you should be doing more for your pet? | Never | Rarely | Sometimes | Quite frequently | Nearly Always |
| 15. Do you feel you could do a better job in caring for your pet? | Never | Rarely | Sometimes | Quite frequently | Nearly Always |

**The following questions are related to your concerns and opinions about anticancer therapy.**

| 16. I feel stressed about the time spent on providing anticancer therapy to my pet. | Strongly disagree | Somewhat disagree | Neutral | Somewhat agree | Strongly agree |
| --- | --- | --- | --- | --- | --- |
| 17. I am worried about the potential side effects of drugs on my pet. | Strongly disagree | Somewhat disagree | Neutral | Somewhat agree | Strongly agree |
| 18. I am concerned about the cost of the anticancer therapy. | Strongly disagree | Somewhat disagree | Neutral | Somewhat agree | Strongly agree |
| 19. I am uncertain about the effectiveness of the anticancer therapy on my pet. | Strongly disagree | Somewhat disagree | Neutral | Somewhat agree | Strongly agree |
| 20. I feel stressed about giving the treatment to my pet on time. | Strongly disagree | Somewhat disagree | Neutral | Somewhat agree | Strongly agree |
| 21. Answer only if your pet receives any chemotherapy    I feel stressed about the special handling of my pet’s body secretions after chemotherapy (e.g. saliva, urine, and feces). | Strongly disagree | Somewhat disagree | Neutral | Somewhat agree | Strongly agree |
| 22. Has your overall stress level changed with your pet’s anticancer therapy? | Much decrease | Slightly decrease | No change | Slightly increase | Much increase |

**Supplementary Table 1: Concerns and Challenges Perceived by Caregivers Regarding Anticancer Therapy**

| **Variable** | **Category** | **Number** | **Percentage (%)** |
| --- | --- | --- | --- |
| Concern about time spent | Much decrease | 9 | 16.7 |
|  | Slightly decrease | 8 | 14.8 |
|  | No changes | 11 | 20.4 |
|  | Slight increase | 21 | 38.9 |
|  | Much increase | 5 | 9.3 |
| Potential side effects of anticancer drug | Much decrease | 2 | 3.7 |
|  | Slightly decrease | 3 | 5.6 |
|  | No changes | 7 | 13.0 |
|  | Slight increase | 24 | 44.4 |
|  | Much increase | 18 | 33.3 |
| Cost of anticancer therapy | Much decrease | 3 | 5.6 |
|  | Slightly decrease | 1 | 1.9 |
|  | No changes | 13 | 24.1 |
|  | Slight increase | 20 | 37.0 |
|  | Much increase | 17 | 31.5 |
| Effectiveness | Much decrease | 3 | 5.6 |
|  | Slightly decrease | 12 | 22.2 |
|  | No changes | 9 | 16.7 |
|  | Slight increase | 26 | 48.1 |
|  | Much increase | 4 | 7.4 |
| Punctuality for appointments | Much decrease | 13 | 24.1 |
|  | Slightly decrease | 13 | 24.1 |
|  | No changes | 8 | 14.8 |
|  | Slight increase | 18 | 33.3 |
|  | Much increase | 2 | 3.7 |
| Special handling of drug | Much decrease | 14 | 31.1 |
|  | Slightly decrease | 10 | 22.2 |
|  | No changes | 11 | 24.4 |
|  | Slight increase | 8 | 17.8 |
|  | Much increase | 2 | 4.4 |
| Overall stress | Much decrease | 3 | 5.6 |
|  | Slightly decrease | 6 | 11.1 |
|  | No changes | 13 | 24.1 |
|  | Slight increase | 28 | 51.9 |
|  | Much increase | 4 | 7.4 |
